# Sex-Related Aspects in Diabetic Kidney Disease—An Update

**DOI:** 10.3390/jcm12082834

**Published:** 2023-04-12

**Authors:** Ivonne Loeffler, Nadja Ziller

**Affiliations:** Department of Internal Medicine III, Jena University Hospital, Friedrich Schiller University, 07747 Jena, Germany

**Keywords:** diabetic kidney disease, DKD, sex differences, gender, sex hormones, transforming growth factor beta 1, TGF-β1

## Abstract

Differences between the sexes exist in many diseases, and in most cases, being a specific sex is considered a risk factor in the development and/or progression. This is not quite so clear in diabetic kidney disease (DKD), the development and severity of which depends on many general factors, such as the duration of diabetes mellitus, glycemic control, and biological risk factors. Similarly, sex-specific factors, such as puberty or andro-/menopause, also determine the microvascular complications in both the male and female sex. In particular, the fact that diabetes mellitus itself influences sex hormone levels, which in turn seem to be involved in renal pathophysiology, highlights the complexity of the question of sex differences in DKD. The major objective of this review is to summarize and simplify the current knowledge on biological sex-related aspects in the development/progression but also treatment strategies of human DKD. It also highlights findings from basic preclinical research that may provide explanations for these differences.

## 1. Introduction

The influence of biological sex differences on human disease has long been underestimated and underresearched. Until recent decades, the vast majority of clinical research was conducted with predominantly male participants. In addition, preclinical research using animal models has almost exclusively examined male animals. Despite this limited approach, it was often (and sometimes still is) assumed that research findings and medical treatments developed from those findings apply to the entire population [1]. However, the resulting lack of understanding limits the ability to treat with targeted and patient-centered therapies. This can have life-threatening consequences for many serious conditions, such as cancer or cardiovascular disease. 

In this review, we focus on the impact of biological sex on diabetic kidney disease (DKD) and therefore use the term “sex” rather than “gender,” which is a social–cultural construct. We follow NIH guidelines that categorize sex as male or female, although variations exist [1]. 

DKD is a secondary disease of type 1 and type 2 diabetes mellitus (T1DM and T2DM). This microvascular complication develops in approximately 30% of patients with T1DM and 40% of patients with T2DM [2] and is characterized by the presence of albuminuria and the progressive loss of renal function [3]. Persistent high blood glucose levels in patients with DM lead to the disruption and damage of the microvascular architecture of the kidneys [4]. As a result, small ultrastructural changes occur in the nephron, mainly localized in the glomerulus and proximal tubule compartment [5]. In renal biopsies of clinical patients with DKD, glomerular changes are most frequently observed [6]. Initial changes include thickening of the glomerular basement membrane (stage I), mild mesangial expansion (>25%), glomerular hypertrophy, and mild microalbuminuria (<30–300 mg/d, stage IIa) [5,6]. Progression of DKD increases the risk of cardiovascular disease and is characterized by an increase in albuminuria (macroalbuminuria > 300 mg/d), severe diffuse mesangial expansion, nodular sclerotic changes (Kimmelstiel–Wilson lesion), decrease in glomerular filtration rate, hyalinosis of afferent and efferent arterioles, loss of podocytes, thickening of tubular basement membrane, tubulointerstitial fibrosis/inflammation, and tubular atrophy (stage IIb-IV) [5,6,7]. Signs of tubulointerstitial fibrosis (TIF) include myofibroblast accumulation, excessive extracellular matrix (ECM) deposition, and renal tubule destruction [8,9].

## 2. Sex Differences in Human DKD

In IgA nephropathy and membranous nephropathy, as well as in nondiabetic chronic kidney disease of unknown etiology, a strong significant association between male sex and adverse renal outcome was observed in a meta-analysis [10]. Other multicenter and population-based studies confirmed that the loss of renal function occurs more slowly in women than in men and the female sex is associated with better survival [11,12,13]. Another meta-analysis indicated the opposite, that progression is faster rather than slower in women. However, the authors acknowledged that most of the women in their analysis were of postmenopausal age and their results may not be generalizable to younger women. Thus, the presumed estrogen-mediated protective effect against nondiabetic chronic kidney disease in younger women compared with men of the same age appears to be lost with the menopause [14,15].

Epidemiological studies show that worldwide, 80% of cases of end-stage kidney failure (ESKF) are due to diabetes, hypertension, or a combination of both. The incidence of ESKF in patients with diabetes is up to ten times higher compared to adults without diabetes [16,17].

Although it seems clear that diabetes-induced macrovascular complications, such as coronary heart disease or stroke, are more common in women [18], data on sex and DKD risk are inconsistent. Studies report either a higher risk in men, a higher risk in women, or no significant sex dimorphism [19,20,21,22,23,24,25]. 

### 2.1. Sex Differences in Development, Progression, and ESKF in DKD

There are several reasons for the inconsistency of data on sex and DKD risk. First, there are different equations for calculating eGFR and gold standards, such as lohexol clearance, are often not used when measuring GFR. In addition, criteria for classifying CKD may need to take into account the distribution of GFR by age and sex [26]. Other, and perhaps the most important, reasons for inconsistency include the types of diabetes (T1DM, T2DM, or both) considered in the studies and the endpoints of interest considered (e.g., micro/macroalbuminuria, eGFR, ESKF, mortality). In addition, studies performing separate-sex analysis vary in sample size and length of follow-up and ethnic cohorts. Although many recent papers and guidelines on DKD generally mention male sex as a more invariant risk factor [16,27], the number of review articles analyzing individual studies on sex differences in DKD in more detail and helping to shed light on the literature jungle is increasing. 

In Table 1, we summarize and present in a highly simplified form what Giandalia et al. and Piani et al., recently described in their reviews of the different studies regarding sex differences in DKD. Taking both reviews together, a total of 55 studies were analyzed, distinguishing between studies of subjects with T1DM (25 studies) or with T2DM (16 studies) and studies that included patients with T1DM and patients with T2DM (14 studies) [26,28]. Of these 55 studies analyzed, 49 showed sex differences (with higher risk in men or in women) and 6 studies showed none. While Giandalia et al., list the individual studies and clearly divide the results according to DKD phenotypes, Piani et al., emphasize the underlying number of subjects and length of follow-up in their tables. It is interesting to note that studies demonstrating a higher risk of DKD in men included a total of 180,000 subjects and had an average length of follow-up of 11.4 years, while studies demonstrating a higher risk in women included more than 5 million subjects with an average length of follow-up of 8.2 years [26].

There are already different study data regarding the development of DKD and the development of micro/macroalbuminuria (Table 1). For example, there is a greater risk for women in the development of DKD for African Americans and Pima Indians with T2DM [29,30]. Regarding the development of micro/macroalbuminuria, the literature is in relative agreement that the male sex is strongly associated with it in both T1DM and T2DM [19,20]. However, there are also a few studies that identified the female sex as a risk factor for the development of microalbuminuria, but surprisingly only in women with a shorter duration of diabetes. This risk decreased again with increasing duration of diabetes [28,31,32].

When looking at the progression of DKD (e.g., by decline in GFR), the data situation is more balanced (Table 1). Especially in studies with patients with T1DM, male as well as female sex is equally often reported as a risk factor [26,28,33,34,35,36,37]. Some studies at ages 18–49 years again have shown no sex differences in DKD progression [38,39,40]. In T2DM, a few more studies have reported faster progression of DKD in women [26,28]. 

When analyzing the studies regarding the influence of sex on ESKF, it is clear that in T1DM, the male sex is the risk factor, whereas in T2DM it is the female sex (Table 1). 

The lower survival rate of individuals with diabetes-related CKD, compared with individuals without CKD, is primarily due to the increased risk of concomitant morbidity associated with CKD, particularly cardiovascular disease. The lack of high-quality population-based studies with validated measures of CKD is the main reason why large differences in the epidemiology of CKD have been observed in populations with diabetes worldwide [16]. For example, women in Israel and Sweden have higher mortality rates if their T1DM developed in childhood, before puberty [41,42]. For men with T2DM and low testosterone levels, testosterone replacement therapy showed reduced mortality [43,44,45].

### 2.2. Factors That May Influence Sex Differences in DKD

**Figure 1:**
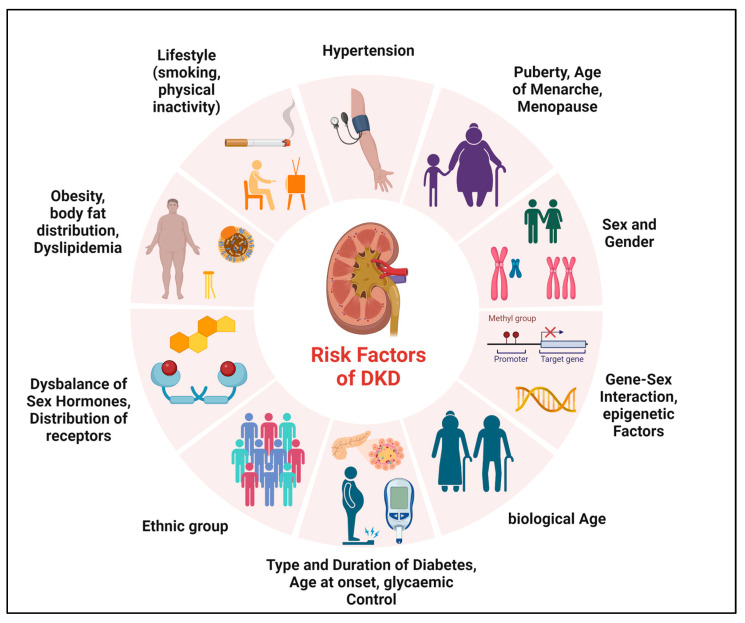
Determinants of sex differences in DKD. Various factors may be determinants of sex differences in the development and progression of DKD.

Diverse factors may be determinants of sex differences in microvascular complications of T1DM and T2DM, which may be unchangeable (such as sex, biological age, and genetic predisposition) or influenceable (such as smoking, physical activity, or glycemic control) (Figure 1). The onset and duration of DM, puberty, or menopause also appear to play a major role in sex differences [12,22,32,46,47,48,49,50].

First and foremost, there are already sex differences in the development of diabetes. T2DM is diagnosed more often at a younger age and lower BMI in men, but the predominant risk factor, obesity, is more common in women [50]. Consistent with the analyses of this work, many studies have shown that women have higher body weights than men at diagnosis of T2DM [51,52]. In addition, newly diagnosed T2DM (>40 years) shows a positive association between small body size and the development of DKD in women [32,53]. There is also evidence that androgen acts directly in peripheral adipose tissue to promote insulin resistance [54]. This is evidenced, for example, by reduced insulin receptor autophosphorylation, decreased expression, and translocalization of the insulin-sensitive glucose transporter, and disruptions in insulin signaling pathways [54]. In contrast, premenopausal women have higher insulin sensitivity compared to postmenopausal women and estradiol has been shown to be protective against insulin resistance [55]. These data indicate that sensitivity to insulin in DM is influenced by sex hormones. Furthermore, the distribution of sex hormone receptors (estrogen and androgen) in subcutaneous adipose tissue is also different in men and women [56,57]. Thus, sex and sex hormones influence adipocyte development, adipogenesis, gene expression profiles responsible for insulin resistance, and lipolysis [56]. 

The quality of glycemic control in patients with T1DM also interacts with sex to determine renal prognosis. Interestingly, in one study, researchers found that among study participants who showed “good” metabolic control, females were more likely to develop DKD, while among participants with “poor” metabolic control, this likelihood was higher in males [12,22,47,58].

Studies have been able to demonstrate that the manifestation of diabetes disease differs in the sexes and the age of onset of DM, especially T1DM, plays an important role in sex differences in DKD risk [42,49,59]. While females are at a higher risk of developing microalbuminuria and even have a higher mortality rate if T1DM occurred in childhood [42], males are at higher risk for it if T1DM occurred with or after puberty [49]. A clear association has been found between higher testosterone levels in younger men and the development of microalbuminuria [60]. This association cannot be shown in an older population of patients with T1DM, reinforcing the concept that what happens in the early phase of diabetes has implications for events many years later [49,61].

There also appears to be a relationship between the onset of menarche and the risk of T1DM-induced microvascular complications: Women with menarche delayed more than 2 years had a 2.3-fold higher risk of DKD (as well as retinopathy) than women with menarche at the average age [62].

The literature on whether and to what extent the sex hormones estradiol and testosterone play a role in DKD is similarly confusing as that on sex differences in the DKD phenotype and is still controversial. However, there is now relative agreement that diabetes leads to an imbalance of sex hormones in both sexes [32,45,48,49,50,61,62,63,64,65,66,67,68,69,70,71,72,73,74,75,76,77,78,79,80,81,82]. The vast majority of studies document that in men with T1DM or T2DM, estradiol levels increase, while testosterone levels decrease, although there are also T1DM data showing increased or unchanged testosterone (reviewed in [48]). In women, DM results in reduced or unchanged estradiol levels and increased testosterone or similar testosterone levels to non-diabetic controls (reviewed in ([48]). However, in postmenopausal women with T2DM, estradiol levels are elevated [32], which, together with the accelerated progression of DKD, may suggest a potential adverse effect of estradiol in the presence of DM.

It is not yet conclusively understood how testosterone and estrogen levels and their respective receptors relate to the progression of DKD in both sexes [32,48,49,50,63].

It is conceivable that an estradiol-mediated mechanism exacerbates the reduction in circulating testosterone in T2DM. This assumption is based on the fact that T2DM in men is associated with increased estradiol levels and that independent studies have shown that activation of the G protein-coupled estrogen receptor in isolated Leydig cells as well as in human testes can downregulate testosterone production [82].

A human study from Finland underlines that not as expected high testosterone levels in diabetic men are the cause of DKD, but that T1DM just leads to reduced serum testosterone concentrations, and that even with the progression of renal damage from micro- to macroalbuminuria, the reduction in testosterone is enhanced [61]. On the other hand, increased testosterone levels are detectable in premenopausal women with T2DM and are associated with insulin resistance and microvascular sequelae [48]. High androgen levels in diabetic women lead to susceptibility to microvascular damage, as DKD can do [82]. 

There is also a large body of research looking at genetic factors that may influence sex differences in DKD. Epidemiological studies have revealed familial clustering of DKD in both types of diabetes as well as a relevant influence of ethnic background [28]. The effects of sex chromosomes as well as the influence of gene–sex interactions with multiple susceptibility genes for DKD have been investigated and recently analyzed by Giandalia et al., for a review [28]. Among others, sex–gene interactions were found for a variant in the angiotensin gene or in the angiotensin II type 1 receptor gene and were described for genes implicated in inflammation and oxidation [28]. Sex differences were also found for variants in the carnosinase gene, CNDP1, on chromosome 18q [28]. A CNDP1 polymorphism associated with low CN1 activity correlates with a significantly reduced risk for DKD, especially in women with T2DM [83].

### 2.3. Data on Possible Underlying Mechanisms in Human DKD

There are a number of mechanisms that are causative for sexual dimorphism. These include mechanisms of hemodynamics (hyperfiltration and the renin–angiotensin–aldosterone system (RAAS)), or in oxidative and substrate stress metabolism, and also the interaction of sex hormones with the signal transduction of TGF-β1 (transforming growth factor beta 1), the main mediator of DKD development and progression, is already known.

As previously described, estradiol can downregulate testosterone production via activation of its receptor [82]. Sex-hormone-related mechanisms are also causal for the gender-specific differentiation of the RAAS, namely that men have a higher RAAS activity than women. While androgens can cause renal vasoconstriction through increased RAAS activity, estradiol on the one hand promotes higher angiotensinogen levels and the ACE2-angiotensin-1–7 axis and on the other hand reduces angiotensin-converting enzyme activity, renin levels, angiotensin II receptor type 1 (AT1R) density, aldosterone secretion, and angiotensin II activity [26].

In the case of oxidative stress, which is an essential pathophysiological feature of DKD, there are indications of sex differences to the disadvantage of the male sex (higher level of oxidative stress in men). The sex hormones play a regulating role in this context: estradiol acts as an antioxidant and androgens increase oxidative stress. Specifically in the kidney, hyperglycemia induces intracellular reactive oxygen species (ROS) in the renal mesangium and tubule cells. Advanced glycation end products (AGEs) and the cytokines TGF-β1 and ANGII are involved in this process. The ROS, in turn, are able to subsequently upregulate extracellular matrix expression via the transcription factors Nuclear Factor Kappa B (NF-κB) and Activator protein-1 (AP-1), which can lead to tubulointerstitial fibrosis (reviewed in [26]).

TGF-β1 is a key factor for pathophysiological processes in DKD. In both type 1 and type 2 diabetes, increased tubular and glomerular TGF-β expression is found in the early as well as late phase of the disease [84]. TGF-β-mediated effects influence the pathology of mesangial cells, podocytes, and endothelial and tubular cells. This leads to cell proliferation, hypertrophy, and apoptosis, and further to inflammation, glomerulosclerosis, and tubulointerstitial fibrosis [84]. By binding and activating its receptor, TGF-β induces a variety of signaling pathways, including both the classical SMAD pathway, which results in the transcription of target genes, and SMAD-independent pathways, such as Ras, JNK, p38, and PI3K [84]. Through TGF-ß1 signaling pathways, the cell has versatile capabilities to control developmental programs autocrine and paracrine, but on the other hand, dysfunctions in this fine-tuned signaling can lead to severe diseases such as the development of DKD [85]. Studies indicate that an important underlying mechanism by which sex hormones mediate their effects in DKD is through the regulation of TGF-β1 [86]. Estrogen can bind SMAD2/3 proteins and inhibit the TGF-β1-induced accumulation of extracellular matrix through activation of the estrogen receptor [86,87]. Another work demonstrated that estradiol can influence TGF-β1-mediated CTGF expression [88]. Regarding the influence of testosterone on TGF-β1, one work was able to show that before puberty there are almost no differences between the two sexes, but after puberty, a threefold higher TGF-β1 production prevails in females than in males, with the activation of latent TGF-β1 dominating in the male sex [89]. Accordingly, it is possible that after puberty, there is much more efficient TGF-β1 activation in males than in females, and in the female sex, the lower activation rates are compensated for by higher basal TGF-β1 levels [89].

### 2.4. Sex Aspects in Pharmacological Studies for Prevention and Treatment of DKD

**Figure 2:**
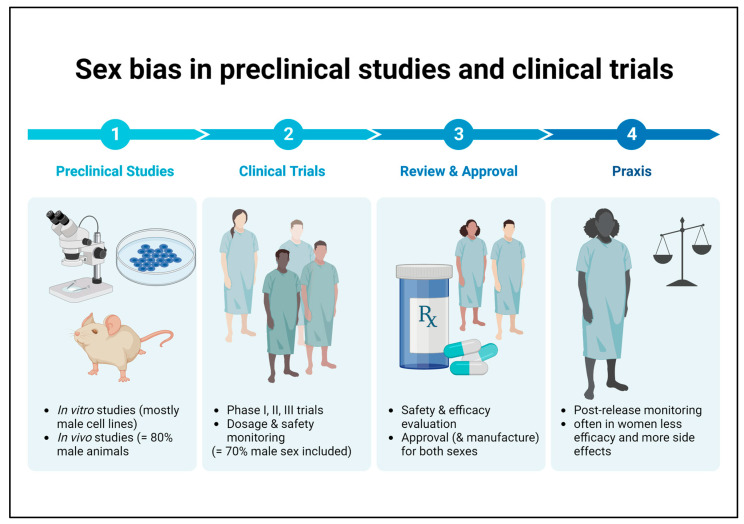
Sex bias in basic preclinical research and clinical trials. Male bias in animal studies as well as in human clinical trials may lead to reduced efficacy or harmful side effects in female patients that remain undetected until the post-marketing observation phase.

It is well known that there is an unequal sex distribution in preclinical research and clinical trials in favor of the male sex. Conducting studies on only one sex and extrapolating the results to the opposite sex can result in reduced efficacy to harmful side effects that may go undetected in the disregarded sex until market launch (Figure 2) [90]. Overall, this is a problem that also applies to medications recommended for patients with DKD, which is why there are no sex-specific guidelines on therapeutic aspects to date. 

Potential therapeutic strategies applicable to the different mechanisms of sexual dimorphism target, for example, sex hormone imbalance, hemodynamic alterations, oxidative stress, or disturbances in water–electrolyte homeostasis and channels [26].

Fortunately, there are more and more editorial provisions from science journals for sex analyses. Since 2016, the SAGER (Sex and Gender Equity in Research) guidelines have been developed by the Gender Policy Committee of EASE (European Association of Science Editors). The SAGER guidelines emphasize strictly separating research subjects and data analysis by biological sex and gender, revealing significant differences even when there were not expected to be any [90]. The relatively recent examples provided below illustrate that these guidelines have not yet been implemented extensively and that a rethinking of study design is urgently needed.

#### 2.4.1. Medications with Primary Reno-Protective Action

Most spironolactone, eplerenone, and finerenone trials of combined RAAS blockade with mineralocorticoid receptor antagonists included 65–98% men, and data were not analyzed separately by sex [91]. Moreover, in the two very important studies, FIDELIO and FIGARO, on the long-term effects of finerenone on kidney and cardiovascular outcomes, the overall population was predominately male (70%) [92,93].

In some important studies, although the sexes were equally distributed, the data were not analyzed separately by sex (e.g., the multicenter study of enalapril and losartan in T1DM; the BENEDICT study of the ACE inhibitor trandolapril and calcium channel blocker verapamil in T2DM; the olmesartan study; the captopril study) [94,95,96,97]. The importance of a separate-sex analysis is shown by a study of irbesartan in DKD (Irbesartan in DN Trial). This study, which actually analyzed data by sex, showed that the progression of DKD is more rapid in women than in men and that women benefit less from treatment than men [32,36]. 

Blockade of endothelin receptor-A has shown significant antiproteinuric effects, and while the trial of the first endothelin receptor-A antagonist tested in phase 3, avosentan, had to be stopped because of side effects, atrasentan seems more promising. Unfortunately, there is also a clear sex bias to the disadvantage of women in the available studies of endothelin receptor type A blockade, as highlighted in the 2019 multicenter study of the effect of atrasentan in DKD in T2DM published in The Lancet. Here, 971 men vs. 352 women were treated with a placebo and 994 men vs. 331 women were treated with atrasentan [98]. 

#### 2.4.2. Antidiabetic Medications with Reno-Protective Effect

Studies of glucose-reducing agents with reno-protective effects, such as the GLP1 (glucagon-like peptide 1) receptor agonists and sodium–glucose transporter 2 (SGLT2) inhibitors, also show a clear sex bias. In a 2017 multicenter study of liraglutide, 64% of the approximately 4600 patients with T2DM were men, and in a study from Denmark, as many as 84% of the patients with T2DM treated were men (reviewed in [99]). 

A 2019 Italian meta-analysis of seven trials involving 56,004 patients with T2DM treated with lixisenatide, liraglutide, exenatide, albiglutide, dulaglutide, and semaglutide did not report the proportion of men and women included or the effects of treatment on each sex [100]. 

In addition to their antihyperglycemic properties, SGLT2 inhibitors also show protective renal effects. They affect hemodynamics, oxidative stress, water–electrolyte homeostasis, and disruption in adiponectin [26]. Except for the CANTATA-SU study in which equal numbers of women and men with T2DM were treated with canagliflozin and glimepiride for nearly 1 year, the studies of empagliflozin (EMPA-KIDNEY, EMPA-REG OUTCOME), dapagliflozin (DAPA-CKD), and canagliflozin (CREDENCE) either included two-thirds men or did not report [99,101,102,103,104,105]. A recent meta-analysis showed that reductions in major adverse cardiac events with SGLT2 inhibitors were lower in women with diabetes than in men with diabetes [106]. Interestingly, in animal studies, higher expression of SGLT2 was found in female rats than in male rats [107]. To what extent the effects shown in the clinical studies can be explained by a possible sex-specific expression of SGLT2 also present in humans certainly requires more intensive research.

## 3. Findings from Preclinical Research Regarding Underlying Mechanisms

Animal experimental research also continues to use more male than female subjects. Although increasing attention has been paid to the female sex for some years, the proportion of male animals in preclinical studies is still up to 80%. This is reflected in the fact that even here, there is still very little knowledge about sex differences. Therefore, similar to human studies, the data on the role of sex in the development and progression of DKD in animal studies are inconclusive. Studies have either been performed only in male or only in female animals, or the hormone status has not been fully determined, namely estradiol and testosterone in both sexes [48].

There are now a number of rodent models of T1DM and T2DM, although the lines available today do not develop all the signs of human DKD according to the Animal Models of Diabetic Complications Consortium (AMDCC) criteria [108]. For example, streptozotocin (STZ)-induced diabetes is a recognized model for T1DM, whereby this cytotoxic glucose analog (STZ) destroys pancreatic ß-cells, resulting in absolute insulin deficiency, hyperglycemia, and weight loss. The animals exhibit a wide spectrum of renal diabetic pathophysiology, such as kidney enlargement, diffuse mesangial expansion, glomerular hypertrophy, basement membrane thickening, ECM accumulation, glomerulosclerosis, and upregulation of TGF-β1 mRNA and protein expression [109,110]. The db/db mouse model of leptin deficiency is currently the most widely used mouse for modeling DKD in settings of T2DM [111]. This has a point mutation in the leptin receptor gene, which leads to obesity, insulin resistance, and infertility. Animals develop obesity and elevated blood glucose levels at approximately 6–8 weeks of age, and by 24 weeks of age, signs of DKD have developed (comparable to moderate- to long-term diabetes mellitus in humans) [112,113]. The underlying genetic background is susceptible to diabetic complications such as nephropathy, and DKD in these mice manifests in albuminuria, podocyte loss, and mesangial matrix expansion. Although these mice do not develop progressive renal insufficiency and therefore fail to recapitulate later and morphologically advanced features of DKD, the db/db mice are a good model of early changes in human DKD [110,111].

In our study of DKD in the T2DM db/db mouse published in 2020, we determined the status of circulating sex hormone concentrations and protein expression levels of sex hormone receptors in the kidneys in both sexes and associated them with DKD [114]. On the one hand, our data provide a complete picture of the imbalance of sexual hormone levels and expression of their receptors in both sexes in the presence of T2DM. However, they also show that there is a complex statistical interaction between sex and T2DM in both plasma estradiol levels and estrogen receptor (ER) expression. In addition to the expected basal differences between males and females, diabetes lowers plasma testosterone in males and plasma estradiol and ER expression in females. In addition, diabetes increases estradiol and ER expression in males [114].

However, how levels of testosterone and estrogen and their respective receptors relate to disease progression in both sexes remains largely unexplained. Indeed, there is some evidence of a paradigm shift away from the traditional simplistic assumption that testosterone is “bad” and estradiol is “good”. For example, it has long been assumed that estrogens inhibit TGF-β1 production, whereas androgens promote TGF-β1 transcriptional activity [115]. Relatively few studies have addressed the sex-specific regulation of the signaling pathways of TGF-β1 and its counterpart BMP7 [114,116].

An animal study revealed a very interesting relationship between sex and the basal TGF-β system in the kidney [117]. Before puberty, no significant differences were seen in active TGF-β1 in either sex, but after puberty, up to a 3-fold increase in TGF-β1 production was observed in females, whereas in males, activation of latent TGF-β1 predominated. This suggests that in males, TGF-β activation becomes more efficient after puberty and females compensate for their reduced activation efficiency by increasing total TGF-β levels [117].

Other studies have shown that orchiectomized diabetic animals with low testosterone replacement therapy had lower TGF-β1 levels than castrated diabetic males without testosterone replacement, but diabetic males with high-dose testosterone administration after orchiectomy had higher TGF-β levels than castrated diabetic males without testosterone replacement [118]. This indicates that concentration plays a crucial role in this regard: high concentrations of dihydrotestosterone (DHT), the most biologically active form of testosterone, stimulate TGF-β1 production and promote DKD in animal models, while a low concentration of DHT, surprisingly, lowers renal TGF-β1 levels and significantly improves the expression of DKD [118]. However, this study by Xu et al., was performed only with male animals, so the question of whether female diabetic animals with altered DHT levels would respond similarly or differently with renal TGF-β expression remains unanswered.

In our study, the holistic approach, i.e., beyond single-group comparisons, revealed that the sex factor significantly influences the effect of diabetes on the expression of the acute phase protein SAA, the profibrotic transcription factor Snail1, and the TGF-β1/BMP7 ratio. In a one-way analysis of expression patterns in intergroup comparisons, some common features become apparent: first, basal expression is significantly higher in female healthy kidneys than in males. Second, the diabetes-dependent upregulation evident in males is less or no longer detectable in females. Third, as a result of the first two points mentioned, no sex differences are detectable in diabetic mice [114]. This may provide an explanation for the conflicting literature data, as single comparisons of diabetic males and females or the influence of diabetes in only one sex may lead to incorrect conclusions if basal levels are unknown or not included. Subsequent ex vivo stimulations of renal tissue with a combination of sex hormones and TGF-β1 revealed differentially regulated expression of the fibrosis factor CTGF (connective tissue growth factor) in both sexes, which additionally reversed as a function of TGF-β1 concentration. The key findings are: (1) depending on sex, DHT influences TGF-β1 effects in opposite ways; (2) in the presence of a low concentration of TGF-β1, DHT has no or rather a lowering effect on TGF-β1-induced CTGF expression in male kidney tissue but an increasing effect in female kidney tissue; and (3) in the presence of high-dose TGF-β1, it is just the opposite: DHT enhances TGF-β1-induced CTGF mRNA in male tissue and has no or rather an inhibitory effect in female kidney tissue [114].

The so-called aromatization of androgens is the last step in the biosynthesis of estrogens. Here, androstenedione is converted to estrone (E1) and testosterone to estradiol (E2). The key enzyme that catalyzes this step is the aromatase CYP19A1, also called estrogen synthase [119]. Increased conversion appears to contribute to the low testosterone levels in diabetic men [120]. In STZ-induced type 1 diabetic rats, CYP activity was shown to be increased in males and decreased in females. In addition, a combination of the inhibition of CYP19 aromatase activity and DHT supplementation was shown to attenuate diabetes-induced renal injury in male rats [121]. This suggests that increased estradiol levels promote DKD. Indeed, blockade of estradiol synthesis in male STZ-induced rats attenuated diabetes-associated renal damage [121]. Further animal studies corroborate a possible negative influence of estrogen signaling, as female transgenic mice lacking estrogen receptor alpha are also protected from diabetes-associated albuminuria and glomerulosclerosis [122].

In turn, other animal experimental approaches showed that complete knockout of the estrogen receptor in nondiabetic mice or ovariectomy of female diabetic rats was associated with increased renal TGF-β1 expression [86]. However, estradiol supplementation of ovariectomized rats or estradiol treatment of diabetic mice resulted in normalization of TGF-β1 expression [86,123].

## 4. Conclusions and Future Directions

Sex is a critical factor in biomedical research. Sex differences in DKD have been described for the development as well as for the progression of and terminal renal failure. Here, depending on the stage of the disease considered, male and female sex crystallize once as a risk factor, which may additionally differ in both types of diabetes. A large number of determinants and molecular factors that promote or inhibit the development and progression of DKD depending on sex have now been identified. Most importantly, events in the early phase of diabetes mellitus have implications for events many years later. Pharmacologic studies of the prevention and treatment of DKD, including those of GLP1 agonists and SGLT2 inhibitors, still have a disproportionate number of men. In addition, however, sex-hormone-dependent changes in expression levels of receptors and transporters have already been described in nondiabetic kidneys, which could have an impact on efficacy as a function of sex. In the spirit of individualized medicine, further sex-specific studies on the possible mechanisms of DKD development and progression, both in animal models and in patient collectives, are urgently needed.

## Figures and Tables

**Table 1:** Summary of analyses by Giandalia et al. [28] and Piani et al. [26] of 49 studies showing sex differences in DKD. Multiple comparisons between albuminuria, eGFR, and ESKF are possible. In each case, the percentage sex distributions indicate the higher risk for sex in the phenotype considered.

Type of Diabetes (Studies’ Number)	Albuminuria		Low eGFR		ESKF	
	Male	Female	Male	Female	Male	Female
T1DM (21)	64.7%	35.3%	50%	50%	85.7%	14.3%
T2DM (15)	71.4%	28.6%	38.5%	61.5%	42.9%	57.1%
T1DM/T2DM (14)	80%	20%	44.5%	55.5%	20%	80%

Abbreviations: eGFR, estimated glomerular filtration rate; ESKF, end-stage kidney failure; T1DM, type 1 diabetes mellitus; T2DM, type 2 diabetes mellitus.

## Data Availability

No new data were created or analyzed in this study. Data sharing is not applicable to this article.
